# Supplementary material for: Comparison of Methods for Incorporating Related Data When Developing Clinical Prediction Models: A Simulation Study
Source: Biom J. 2026 Aug 27;68(5):e70166. doi: 10.1002/bimj.70166 (PMC13520826; doi:10.1002/bimj.70166)
Supplement: Supplementary file 2 [file BIMJ-68-e70166-s001.zip › third submission code review - R2/reproducibility_supplement/results/case_study/FIGURE7-Performance Metrics of SWEDEHEART Case-Study.pdf]

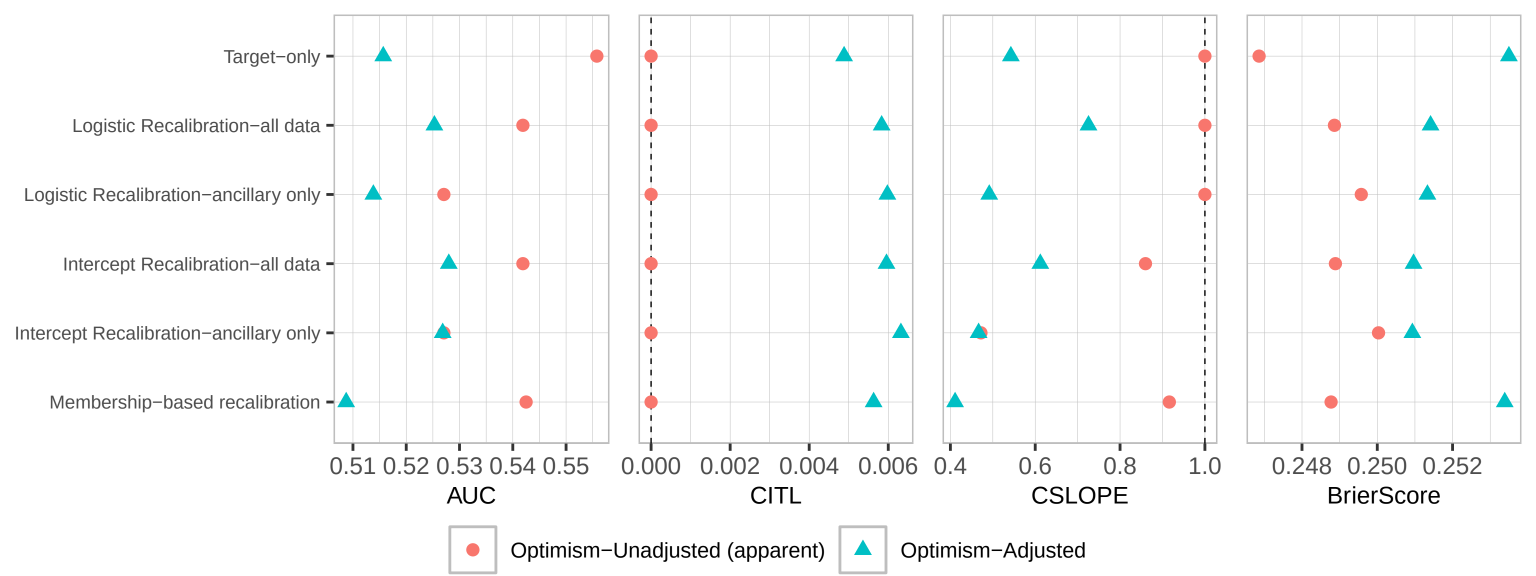

Target−only
Logistic Recalibration−all data
Logistic Recalibration−ancillary only
Intercept Recalibration−all data
Intercept Recalibration−ancillary only
Membership−based recalibration
0.51 0.52 0.53 0.54 0.55
AUC
0.000 0.002 0.004 0.006
CITL
0.4 0.6 0.8 1.0
CSLOPE
0.248 0.250 0.252
BrierScore
Optimism−Unadjusted (apparent)
Optimism−Adjusted
